# Integrated agronomy of pea (*Pisum sativum L.*): a review on cultivation, harvesting, and storage for sustainable agriculture

**DOI:** 10.3389/fpls.2025.1670445

**Published:** 2025-09-30

**Authors:** Honglei Zhang, Hongyan Sun, Zhong Tang, Guoqiang Wang

**Affiliations:** ^1^ School of Agricultural Engineering, Jiangsu University, Zhenjiang, China; ^2^ School of Electrical Engineering, Nanjing Normal University Taizhou College, Taizhou, China; ^3^ School of Electrical Engineering, Nanjing University of Aeronautics and Astronautics, Nanjing, China; ^4^ School of Agricultural Engineering, Jiangsu Agri-animal Husbandry Vocational College, Taizhou, China

**Keywords:** pulse crop, nitrogen fixation, post-harvest loss, food security, climate resilience

## Abstract

Peas (*Pisum sativum L.*) are a cornerstone of sustainable agriculture, yet their potential is limited by fragmented agronomic practices. This review provides an integrated synthesis of advancements across cultivation, mechanized harvesting, and post-harvest storage. Key findings reveal that optimal growth conditions and nanotechnology interventions can significantly enhance abiotic stress tolerance. Mechanized harvesting innovations reduce yield losses by up to 40%, but smallholder adoption and terrain compatibility remain critical challenges. Effective post-harvest strategies, including low-temperature storage and hermetic bags, are crucial for preserving quality. Despite progress, systemic barriers persist. Future research must prioritize interdisciplinary solutions—combining genomics, precision engineering, and farmer training—to unlock the full potential of peas as a keystone crop for sustainable food systems.

## 1 Introduction

The domestication of crops (Trneny et al., 2018; Alseekh et al., 2021) marked a pivotal milestone in human history, enabling stable food production and catalyzing the transition from hunter-gatherer societies to agrarian economies, thereby laying the foundation for modern civilization. Among the earliest domesticated plants, pea (*Pisum sativum*) remains a globally significant rotation and cash crop (Malcolmson et al., 2014; Daba et al., 2025), cultivated in over 90 countries (FAO, 2023). Recent data indicate 7.41 million hectares dedicated to dry pea production and 2.66 million hectares to green peas worldwide, underscoring its agricultural prominence.

Peas have served as a cornerstone of genetic research since Mendel’s pioneering work on heredity (Kuzbakova et al., 2022; Chandel et al., 2023; Sainju and Pradhan, 2024). Beyond their role in science, peas enhance agricultural sustainability through biological nitrogen fixation, which minimizes synthetic fertilizer use and improves soil health (Mirzad et al., 2023). As a rotational crop, they further mitigate pest and disease cycles (Shanthakumar et al., 2022; Mirzad et al., 2023), solidifying their multidisciplinary value across agriculture, medicine, and environmental science (Javed et al., 2021; Raza et al., 2021; Hashim et al., 2022).

Despite extensive research on peas, comprehensive agronomic reviews that bridge the entire production chain remain limited. The growing global demand for plant-based proteins and the increasing pressures of climate change make pea an ideal candidate for sustainable intensification, yet its potential is often unrealized due to fragmented knowledge. Existing literature predominantly addresses specific traits like nutritional profiles (Shanthakumar et al., 2022; Sulima and Zhukov, 2022), breeding for disease resistance (Pheirim et al., 2022; Sinjushin et al., 2022), and varietal classification (Abdel-Aal, 2024; Sun et al., 2024), often in isolation. Critical gaps persist in synthesizing this information into an integrated framework that connects cultivation environments, agronomic practices, harvesting techniques, and post-harvest storage protocols (Boukid et al., 2021; Wu et al., 2023). This fragmentation is a critical barrier to optimizing the pea value chain for sustainable agriculture.

To address these gaps, this review provides a systematic and holistic synthesis of modern pea agronomy. The primary objectives are to: (1) analyze optimal cultivation environments and stress-response strategies, incorporating recent biotechnological advances; (2) evaluate the evolution and current state of mechanized harvesting technologies, identifying key barriers to adoption; and (3) synthesize best practices for post-harvest storage to minimize losses and maintain quality. The novelty of this work lies in its integrative approach, connecting advancements across the entire production chain to highlight synergies and systemic challenges. By synthesizing fragmented knowledge, this review aims to provide a clear framework for future interdisciplinary research and innovation in pea agronomy, ultimately supporting its role in sustainable food systems.

## 2 Methodology

**Figure 1:**
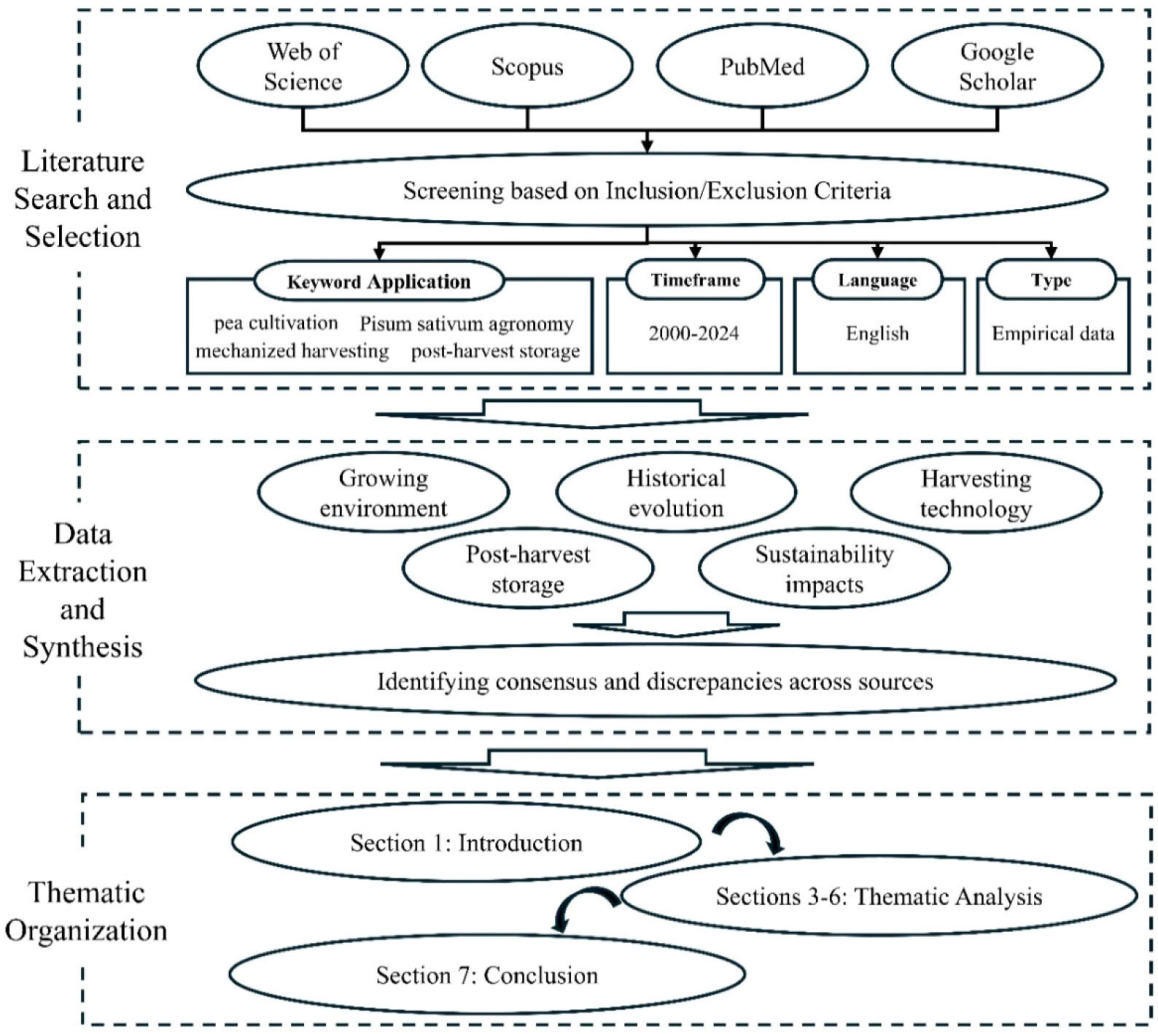
Schematic flowchart of the systematic review methodology.

This review adopts a systematic approach to synthesize existing knowledge on pea (*Pisum sativum L*.) agronomy, focusing on cultivation environments, harvesting technologies, and storage practices. The methodology comprised three stages: (1) Literature Search and Selection, (2) Data Extraction and Synthesis, and (3) Thematic Organization (as summarized in **Figure 1**).

1. Literature search and selection: Peer-reviewed articles, books, and technical reports were sourced from databases including Web of Science, Scopus, PubMed, and Google Scholar. Keywords such as “pea cultivation,” “Pisum sativum agronomy,” “mechanized harvesting,” “post-harvest storage,” and “pea genetic diversity” were employed. Inclusion criteria prioritized studies published between 2000–2024 to emphasize recent advancements, though seminal works (e.g., Mendel’s foundational studies) were retained for historical context. Articles were excluded if they lacked empirical data, focused solely on non-agronomic traits (e.g., pure nutritional analyses), or were not available in English.
2. Data extraction and synthesis: Extracted data were categorized into five themes aligned with the review’s objectives: (i) growing environment, (ii) historical evolution and growth conditios, (iii) development of mechanized pea harvesting technology, (iv) post-harvest storage, and (v) sustainability impacts. Cross-referencing ensured coverage of both field-based studies (e.g., soil management trials) and technological innovations (e.g., CRISPR applications). Discrepancies in findings were resolved by prioritizing consensus across multiple sources.
3. Thematic organization: The synthesized data were structured into seven sections to ensure logical progression. Section 1 contextualizes the historical and socioeconomic role of peas. Sections 2 provides an overview of the methodology used for the synthesis. Sections 3–6 critically evaluate agronomic practices, genetic traits, mechanization, and storage protocols, while Section 7 integrates insights to identify research gaps and future priorities. This structured methodology ensures coherence, minimizes bias, and facilitates interdisciplinary linkages across agronomy, genetics, and engineering.

## 3 Historical evolution and growth conditions

Scholars have proposed various hypotheses regarding the origin of pea (*Pisum sativum L*.), though consensus identifies its domestication in regions spanning western Asia, the Mediterranean, Asia Minor, Transcaucasia, and Ethiopia approximately 10,000 years ago (Pheirim et al., 2022). Wild pea subspecies, including *Pisum sativum subsp*. *elatius*, are distributed across Central Asia, the Near East, and North Africa. Genetic evidence suggests hybridization between these wild populations and early cultivated varieties formed the progenitor of modern pea cultivars. The geographic distribution of wild subspecies strongly supports these regions as primary centers of pea domestication. This chapter synthesizes current understanding of pea’s origin, taxonomic classification, and agronomically significant growth traits.

### 3.1 The species of peas

Peas (*Pisum sativum L*.) have been domesticated for over 6,000 years, with archaeological evidence including 9,000-year-old carbonized seeds from Neolithic sites in Turkey. Ancient Greek and Roman texts further confirm their early cultivation in Europe. Following domestication, peas spread northwestward across southern Europe. Historical records suggest their introduction to India predated Persian and Greek influence in the region.

Initially cultivated for dried seeds, pea consumption shifted during the Middle Ages with the emergence of podded vegetable varieties (Bagheri et al., 2023). Archaeological remains from 9th–11th century Swedish tombs and 18th-century Dutch records document this transition, with vegetable peas introduced to England circa 1760. By the 17th century, peas were extensively cultivated in Europe and introduced to North America (1636) and Oceania via colonial expansion. Historical accounts suggest Silk Road dissemination to China during the Western Han Dynasty (2nd century BCE), facilitating their spread across East Asia.

Taxonomically, peas (*Pisum sativum L.*) belong to the family Fabaceae. Modern cultivated peas (*P. sativum ssp. sativum*) are broadly divided into garden peas (*var. sativum*) and field peas (*var. arvense*), which diverged from their wild progenitor, *P. sativum ssp. Elatius* (Munoz et al., 2017; Salgotra and Stewart, 2022). This domestication process laid the groundwork for critical agronomic developments.

Beyond initial domestication, several key milestones have shaped modern pea agronomy. A pivotal breeding breakthrough in the 20th century was the development of semi-dwarf, semi-leafless cultivars. This innovation drastically reduced lodging, improved light penetration into the canopy, and critically, facilitated the transition to large-scale mechanized harvesting, which was previously hindered by the vining habit of traditional tall varieties. This shift in plant architecture is a cornerstone of modern pea production. Concurrently, global cultivation trends have undergone significant shifts. While historically centered in Europe for dry pea production, the late 20th century saw North America and Australia emerge as dominant, export-focused producers. More recently, there has been a notable increase in cultivation in Asia, driven by demand for fresh vegetable peas, reflecting a diversification of both production systems and end uses.

The white-flowered pea (*Pisum sativum subsp*. *sativum* var. *sativum*), or vegetable pea, produces spherical, wrinkled seeds in yellowish-white to bluish-green hues. Its tender pods are consumed as vegetables, while stems serve as forage and root residues as organic fertilizer. The purple-flowered pea (*Pisum sativum* subsp. *Sativum* var. *arvense*), or grain pea, exhibits purple to red-blue flowers and mottled gray-brown seeds. This hardy, tall-growing variety is suited for large-scale cultivation as fodder or green manure, though its high yield is offset by lower culinary quality.

**Figure 2:**
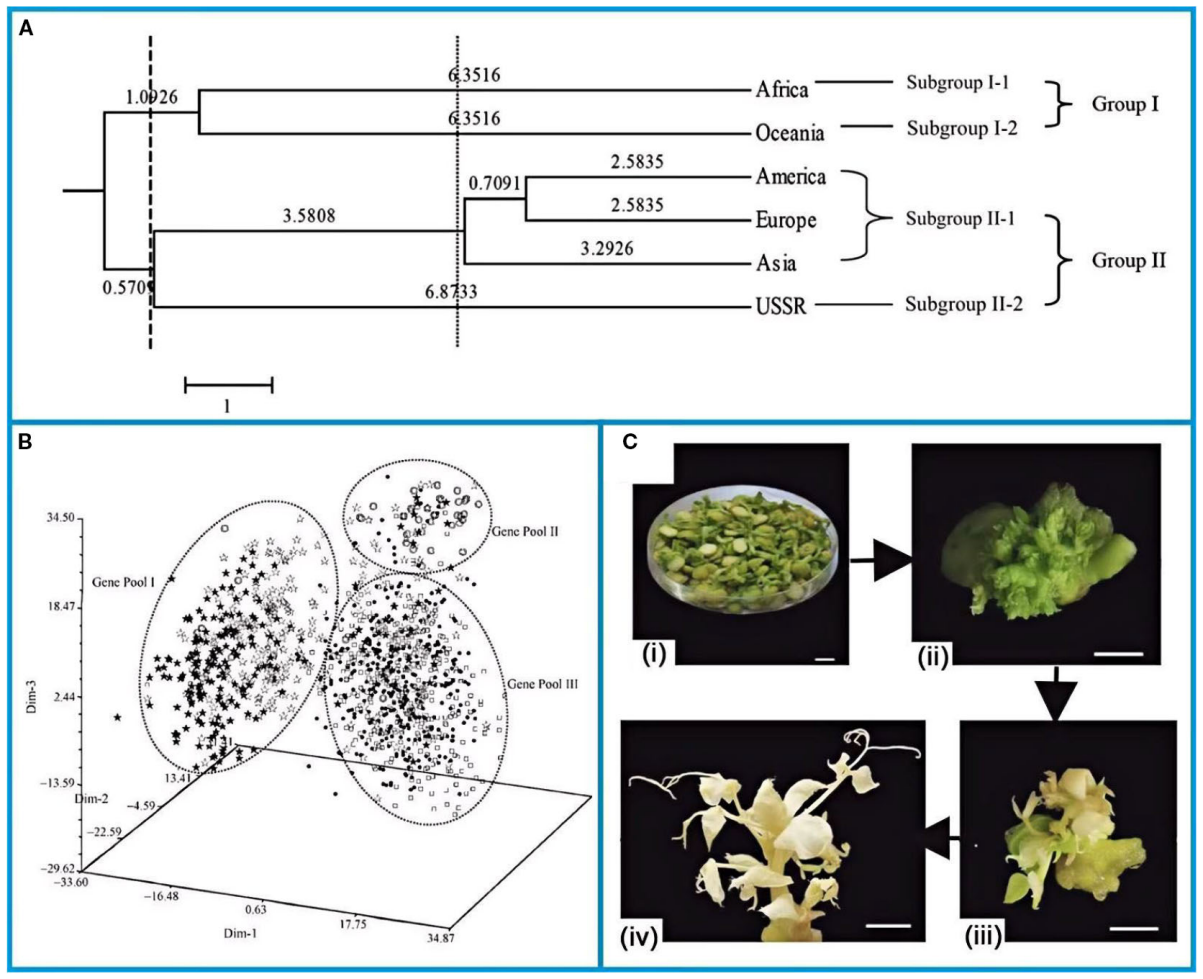
**(A)** Dendrogram of continental groups of pea genetic resources using UPGMA based on SSR analysis (Zong et al., 2008b). **(B)** Three-dimension PCA graph of pea landraces from China using Euclid distance based on SSR analysis (Zong et al., 2008a). **(C)** Flow diagrams of stablegenetic transformation in pea: (i) explants, (ii)clustered buds, (iii) bud elongation, and (iv) the successfully edited albino In additions, peas as a long day c (Li et al., 2023a). The figure was reproduced from Ref (Zong et al., 2008b; Zong et al., 2008a; Li et al., 2023a).with permission from the rightsholder.

Cultivated in China for ~2,000 years, peas are documented in post-Han agricultural texts. China now curates over 5,000 pea accessions, including 1,000 cultivated varieties from 70 nations, with 20% sourced from Australia, the U.S., Europe, and Asia (Delvento et al., 2023). China holds nearly 1,000 cultivated pea species from 70 countries on five continents. Zong et al (Zong et al., 2008b). assessed that genetic diversity of introduced pea germplasm and constructed their core collection using 21 pairs of simple sequence repeat (SSR) primers. The European, Asian and American groups were closely related to each other and had the shortest genetic distances (**Figure 2A**). Therefore, they were grouped into the same clustering subgroups. Despite the fact that the USSR is located on the Eurasian plate and is geographically similar, the Soviet Union entries were separated from the Asian and European groups. The results suggested that the Asian group experienced the highest level of genetic diversity, followed by the European group, and the Oceania group had the lowest level of genetic diversity.

Zong et al (Zong et al., 2008a). furtherreported the genetic diversity of Chinese pea and the genetic relationships among germplasm from different sowing areas and provinces. Among the Chinese pea landraces, three gene pools were identified, which were typified by landraces from Inner Mongolia and Shaanxi (Genepool I), landraces from Henan (Genepool II) and landraces from other provinces (Genepool III) respectively (**Figure 2B**). According to the three-dimensional principal component analysis (PCA), there was little overlap among the three gene pools, and the Nei’s (1978) genetic distances among provinces were 5.159~27.586. The findings suggested that the genetic diversity of Chinese pea local varieties was related to their ecological and geographical distribution.

As a typically self-pollinated plant, pea possesses a narrow genetic base (Jing et al., 2007), which renders it difficult to breed cultivars other than those with excellent agronomic shapes, especially for traits with complex intrinsic associations (Burstin et al., 2015). Based on the above, neither traditional nor modern breeding techniques improved the agronomic traits of pea to a high degree. Gene editing, utilizing engineered nucleases (“molecular scissors”) for targeted gene modifications, offers rapid, precise, and transgene-free improvements, surpassing traditional methods (Gaj et al., 2013; Zhu et al., 2020).

In addition, Clustered Regularly Interspaced Short Stranded Nucleic Acid Repeats (CRISPR)/CRISPR-associated nuclease 9 (Cas9) is extensively employed as the latest generation of tools for gene editing (Bibikova et al., 2002; Li et al., 2011; Manghwar et al., 2019). The Agrobacterium-mediated CRISPR/Cas9 system was successfully developed by optimising the engineering reagents for CRISPR/Cas9 constructs (Li et al., 2023b). The flowchart of stable genetic transformation of pea was shown in the **Figure 2C** (Li et al., 2023a). From left to right, exosomes, poly shoots, shoot elongation and successfully edited albino lines were shown. Pea albino mutants were successfully obtained using this novel system. The bridge between genetic modelling and the modern genetic era has been built through the successful development of pea mutants.

### 3.2 The growth characteristics of peas

Pea is a climbing annual herbaceous plant. When mature, it reaches a height of 0.5–2 meters. The plant is green, smooth, and glabrous. It has 4.0-6.0 leaves. The stipules are larger than the leaflets, cordate in shape, with fine teeth on the lower edge. The leaflets are ovate, about 2.0-5.0 cm in height and about 1.0-2.5 cm in width. The flowers are solitary in the leaf axils or arranged in several racemes. The calyx of the pea flower is campanulate with lanceolate lobes. The corolla comes in various colors depending on the variety, but most are white or purple (Karkanis et al., 2016).

The ovary of pea is glabrous and the style is flattened and bearded inside. The pods are swollen in form and have an elongated oval shape, 2.5–10 cm (length) and 0.7-14.0 cm (width) respectively. The pods are pointed apically, nearly straight dorsally, and have a hard papery endodermis on the inside. The pods contain 2.0-10.0 seeds, round in shape, lime green in color, smooth and wrinkled. The seeds are yellow when dried. In the Northern Hemisphere, the flowering period of peas is from June to July, and the fruiting period is from July to September. In the Southern Hemisphere, the flowering period of peas is usually from May to July, and the fruiting period is from July to September.

**Figure 3:**
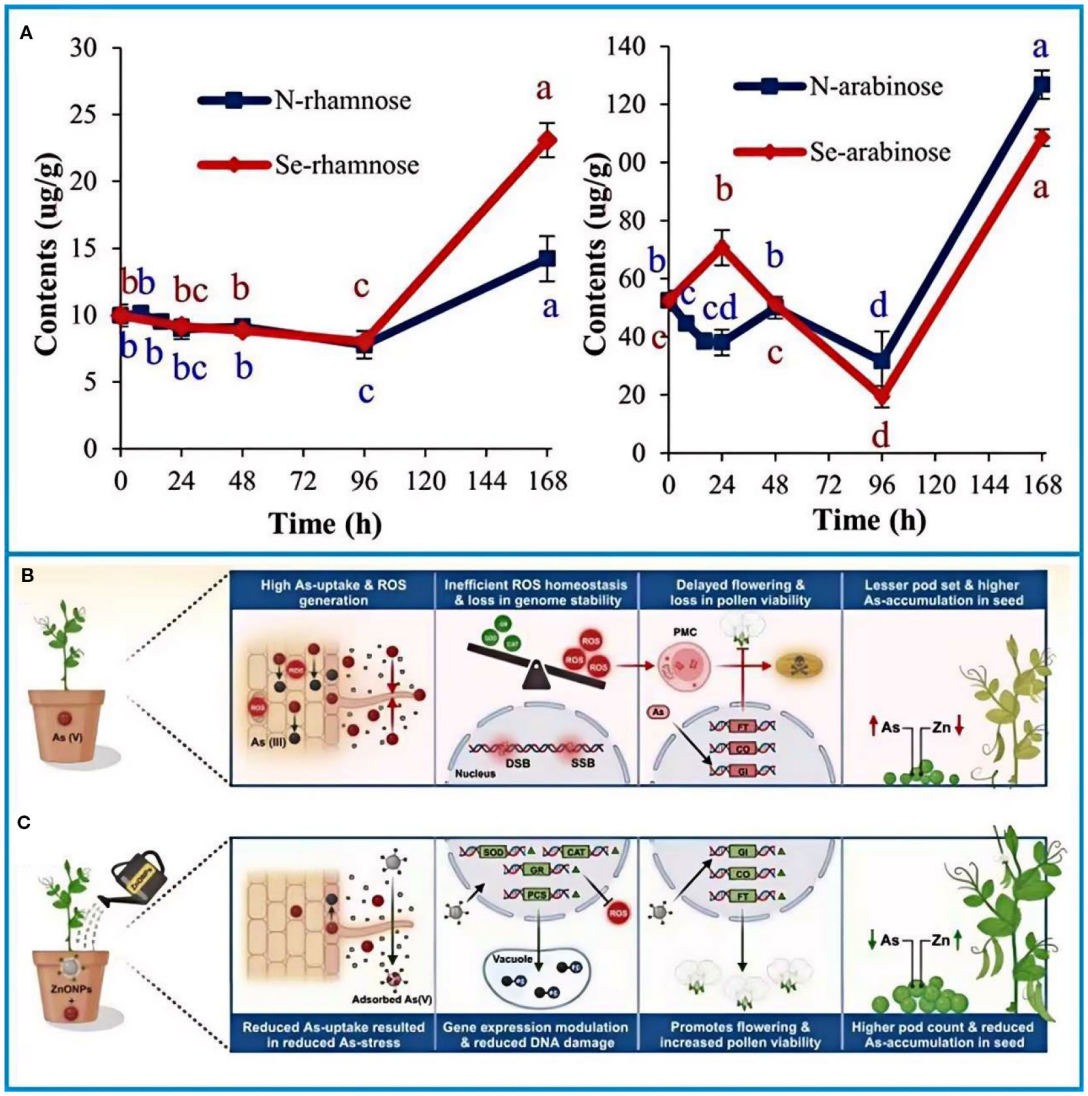
**(A)** Effect of different treatments on sugar content of pea shoots (Xue et al., 2024). **(B, C)** Schematic of zinc oxide nanoparticles rescuing arsenic-toxic peas (Banerjee et al., 2024). The figure was reproduced from Ref (Banerjee et al., 2024; Xue et al., 2024). with permission from the rightsholder.

Germination enhances carbohydrate utilization and nutrient bioavailability in peas (Paucar-Menacho et al., 2010; López-Martínez et al., 2017). Selenium (Se), a vital trace element for plant growth, has been shown to regulate sugar metabolism during early germination (Nikakhlagh et al., 2021). Xue et al (Xue et al., 2024). demonstrated that nano-selenium (30 mg Se/L) significantly increased sugar content in pea shoots after 96 hours of treatment (**Figure 3A**), promoting seed growth by modulating early-stage metabolic processes.

Flowering closely follows the germination of peas. Flowering is a critical stage in the plant lifecycle that determines crop productivity and that is regulated by genetic and photodynamic pathways. Nevertheless, in Southeast Asia soils are enriched with arsenic due to human activities such as mining, overuse of arsenic-containing pesticides, and groundwater contamination of soils (Rahaman et al., 2013; Calatayud et al., 2018; Saha et al., 2021). Arsenic toxicity disrupts pollen viability, reduces germination rates, and induces oxidative stress (Gupta and Bhatnagar, 2015). Recent advances in nanotechnology offer promising solutions. Zinc oxide nanoparticles (ZnONPs), for instance, act as both micronutrients and adsorbents for heavy metals (Ali et al., 2021; Fegade et al., 2023). Studies indicate that ZnONPs mitigate arsenic uptake in peas, enhance rhizobium activity, and improve antioxidant responses, thereby restoring pollen viability and reducing genotoxicity (**Figures 3B, C**) (Banerjee et al., 2024). This rhizosphere nanoremediation strategy highlights the potential of nanomaterials in addressing soil contamination without overemphasizing technical mechanisms.

While peas are generally resilient to zinc deficiency, prolonged overuse of agrochemicals has depleted soil zinc levels in intensive farming systems (Poblaciones and Rengel, 2017). Zinc deficiency manifests as stunted growth, chlorosis, and reduced stress tolerance. Foliar application of ZnSO_4_·7H_2_O (1.0%) at critical growth stages (germination and flowering) has been shown to enhance yield and seed quality (Dhaliwal et al., 2022), underscoring the importance of balanced micronutrient management.

As a crop that has been domesticated by mankind for a long time, peas have had a significant impact on human development. This chapter deals with the origin and classification of peas and discusses the difficulties that may be encountered during the growth of peas and the corresponding solutions. This provides corresponding ideas for the difficulties encountered during pea cultivation and facilitates the large-scale cultivation of peas.

Under organic production conditions, pea growing has several possibilities. Peas can be grown using organic fertilizers like compost and manure to enrich the soil. Crop rotation with non-leguminous plants helps maintain soil health and control pests. Natural predators can be introduced to manage aphids and pea weevils. Additionally, resistant pea varieties can be selected to reduce disease incidence, such as those less prone to PSbMV. This holistic approach promotes sustainable pea cultivation.

## 4 Growing environment

Peas (*Pisum sativum L.*) are cultivated across diverse agroecological conditions, including arid regions, owing to their adaptability (Ram et al., 2021). Notably, peas thrive in nutrient-deficient soils, where they enhance soil health by regulating microbial activity and improving granular structure, positioning them as a valuable pre-crop (Bagheri et al., 2023). As a primary early spring crop, peas are integral to optimizing planting systems through strategies such as intercropping, relay planting, crop rotation, and fallow management (Fortier et al., 2023). Peas play a vital role in promoting agricultural sustainability and enhancing dietary diversity. Consequently, peas hold strong and growing market demand globally. Advancing research on pea cultivation and effective management of pests (e.g., *Acyrthosiphon pisum*, *Bruchus pisorum*, *aphids*) and diseases (e.g., Pea Seed-Borne Mosaic Virus, powdery mildew) is critical to fostering sustainable pea production. This chapter examines optimal environmental conditions for pea cultivation and synthesizes best practices for field management.

### 4.1 Ecological adaptations and abiotic stress responses in pea

As a leguminous species, pea (*Pisum sativum L.*) forms a symbiosis with rhizobia bacteria, enabling it to fix atmospheric nitrogen through root nodulation. The plant develops a taproot system where most lateral roots are concentrated in the top 25 cm of soil, while the primary root can extend up to 1.7 m deep. This structure optimizes both nutrient uptake and soil stabilization. Although rhizobial activity is highest in slightly acidic soils (pH 6.7–7.3), the symbiosis can function across a pH range of 6.5–8.0. Alkaline conditions enhance nitrogenase activity, while acidity reduces nodulation efficiency (Lejeune-Hénaut et al., 2008). The main ecological drivers are summarized in **Table 1**.

**Table 1:** Summary of key ecological drivers.

Factor	Optimal range	Stress thresholds	Adaptive mechanisms
Temperature	15–22 °C (growth)	>25 °C (flowering)	Heat shock proteins, ABA signaling
Soil pH	6.7–7.3	<6.5 or >8.0	Rhizobial symbiosis optimization
Soil Nutrients (N, P, K)	Low initial N; Adequate P and K based on soil tests	Deficiency of N, P, or K; Excess N inhibits nodulation	Symbiotic nitrogen fixation (Rhizobium); Mycorrhizal associations for P uptake; Efficient root transporters
Moisture	70–90% RH (flowering)	<60% RH (pod drop)	FeO-NP priming, ROS scavenging
Photoperiod	>12 h daylight	<10 h (delayed flowering)	Photoreceptor-mediated flowering

Pea’s winter hardiness stems from key molecular adaptations. These include the stable expression of housekeeping proteins and the flexible regulation of stress-response proteins during cold periods (Jing et al., 2007; Zong et al., 2008a; Li et al., 2023a). Germination can begin at a cold 2–5 °C, but emergence is most successful between 14–19 °C. Optimal temperatures for subsequent growth stages are 12–16 °C for vegetative growth and 15–22 °C for flowering and pod development. High temperatures are particularly damaging during flowering; temperatures above 25 °C can cause pollen sterility and ovule abortion, reducing pod set by 20–40% (Cortes and Blair, 2018; Balliu and Sallaku, 2021).

Heat tolerance in pea is strongly correlated with plant architecture. Under heat stress, medium-tall genotypes (80–150 cm) show greater yield stability than dwarf phenotypes. This resilience is attributed to several factors: their grain-filling periods are 7–10% longer, they retain 8–18% more pod nodes, and their overall yield loss is 13–18% lower (Parihar et al., 2023). Key traits for heat resilience include semi-few-leafed morphology, upright growth habit, and enhanced source-sink efficiency (Sadras et al., 2013; Jiang et al., 2018; Tafesse et al., 2019).

**Figure 4:**
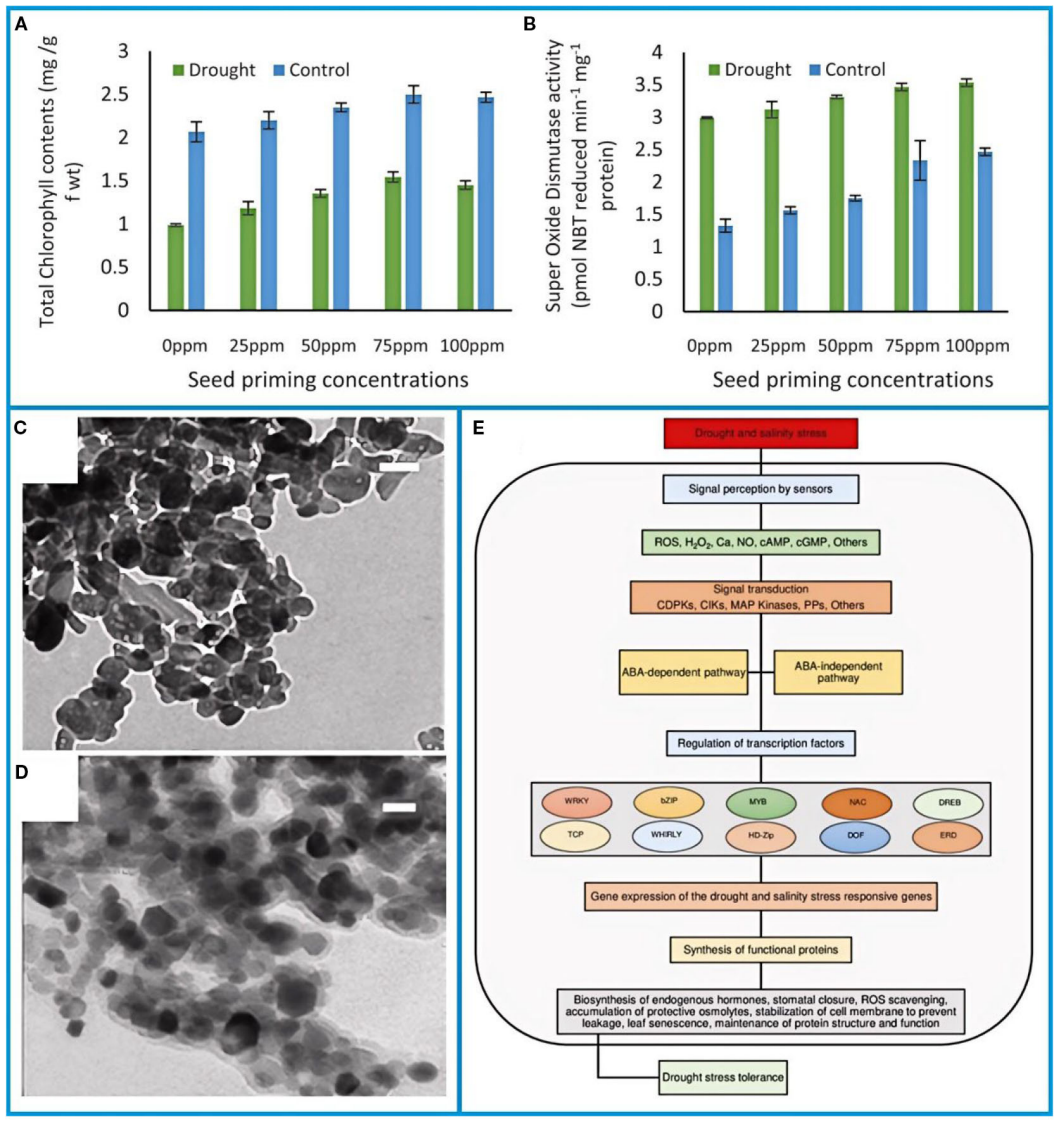
**(A)** Total chlorophyll and **(B)** activities of antioxidant enzymes in pea plants raised through FeO-NPs primed seeds (Mazhar et al., 2023). **(C)** TEM images of ZnO NPs (scale bar, 50 nm) and **(D)** ZnO-Si NPs (scale bar, 20 nm) (Elshoky et al., 2021). **(E)** Schematic representation of drought and salinity stress tolerance mechanism in plants (Demirkol and Yilmaz, 2023). The figure was reproduced from Ref (Elshoky et al., 2021; Demirkol and Yilmaz, 2023; Mazhar et al., 2023). with permission from the rightsholder.

Although seedlings can tolerate brief dry periods, water deficits during the critical flowering stage are highly damaging. Such stress reduces stomatal conductance, chlorophyll content, and photosynthetic efficiency by 30–50%, which in turn accelerates leaf senescence. As a defense mechanism, drought-induced oxidative stress increases the activity of antioxidant enzymes like catalase and peroxidase (**Figures 4A-B**) (Mazhar et al., 2023).

Seed priming with iron hydroxide nanoparticles (FeO-NPs, 75 ppm) mitigates drought impacts by enhancing chlorophyll retention (28.7% increase) and improving soil water-use efficiency (Manzoor et al., 2023). Nanotechnology interventions, including silica-coated ZnO nanoparticles (ZnO-Si NPs), further alleviate salt-drought synergism by reducing ionic toxicity and oxidative damage (**Figures 4C–D**) (Elshoky et al., 2021).

However, while promising, the application of nanotechnology in agriculture warrants a balanced perspective. Critical gaps remain in understanding their long-term ecological impacts, such as nanoparticle accumulation in soil and potential ecotoxicity to non-target organisms. Furthermore, regulatory frameworks for the agricultural use of nanomaterials are still evolving in many regions, creating uncertainty for widespread adoption. Finally, their cost-effectiveness and the scalability of application methods for resource-limited smallholder farmers present significant hurdles that must be addressed before these interventions can be considered broadly sustainable.

Soil and photoperiod factors further modulate adaptation. As a long-day crop, pea requires >12 h daylight for optimal flowering. Photoperiod shortening delays anthesis, induces internode shortening, and promotes abnormal stipule development. High planting density exacerbates light competition, increasing pod abscission by 15–30% due to carbohydrate limitation (Ram et al., 2021).

Pea thrives in well-drained sandy loams with high organic matter (>2%). Waterlogging induces root hypoxia, reducing nodulation and nitrogen fixation by 40–60%. During pod filling, air humidity <60% or temperatures >25 °C accelerates senescence, shortening the maturation period by 5–7 days and lowering yield by 20–35% (Ram et al., 2021).

Combined abiotic stresses (e.g., salinity-drought) disrupt osmotic balance and ROS homeostasis, necessitating multi-tiered molecular responses (**Figure 4E**) (Demirkol and Yilmaz, 2023). ABA-mediated signaling pathways activate stomatal closure, osmolyte biosynthesis (proline, glycine betaine), and antioxidant systems (SOD, APX) to sustain membrane integrity and photosynthetic function (Shamloo-Dashtpagerdi et al., 2022). Screening of 48 pea genotypes identified traits for breeding programs, including elevated pod wall ratios, enhanced ovule retention, and source-sink optimization under stress.

### 4.2 Field management strategies for pea cultivation

Peas (Pisum sativum L.), as cool-season legumes, require precise agronomic practices to achieve high yield and quality. This section systematically outlines key field management strategies, integrating recent scientific advances with practical applications.

#### 4.2.1 Crop rotation and soil preparation

Effective crop rotation is essential for controlling soil-borne diseases and preventing nutrient depletion. To disrupt pathogen cycles and improve soil health, farmers should avoid planting peas in the same field continuously and instead use a 2–3-year rotation with cereal crops like wheat or barley. Proper soil preparation before sowing includes deep plowing (25–30 cm) to improve root penetration and aeration. This step is particularly important because pea roots are shallow and sensitive to soil compaction.

Pre-planting basal fertilization should combine organic amendments (3–4 t/ha decomposed manure) with mineral fertilizers applied at a ratio of N:P_2_O_5_:K_2_O = 5:7:3 (20–25 kg/ha), placed 5 cm below the seed furrow to optimize nutrient availability during germination. Drainage channels (ridge width: 1.2–1.5 m) must be constructed to prevent waterlogging, as pea roots tolerate submersion for only 48–72 hours.

#### 4.2.2 Fertilization management

**Figure 5:**
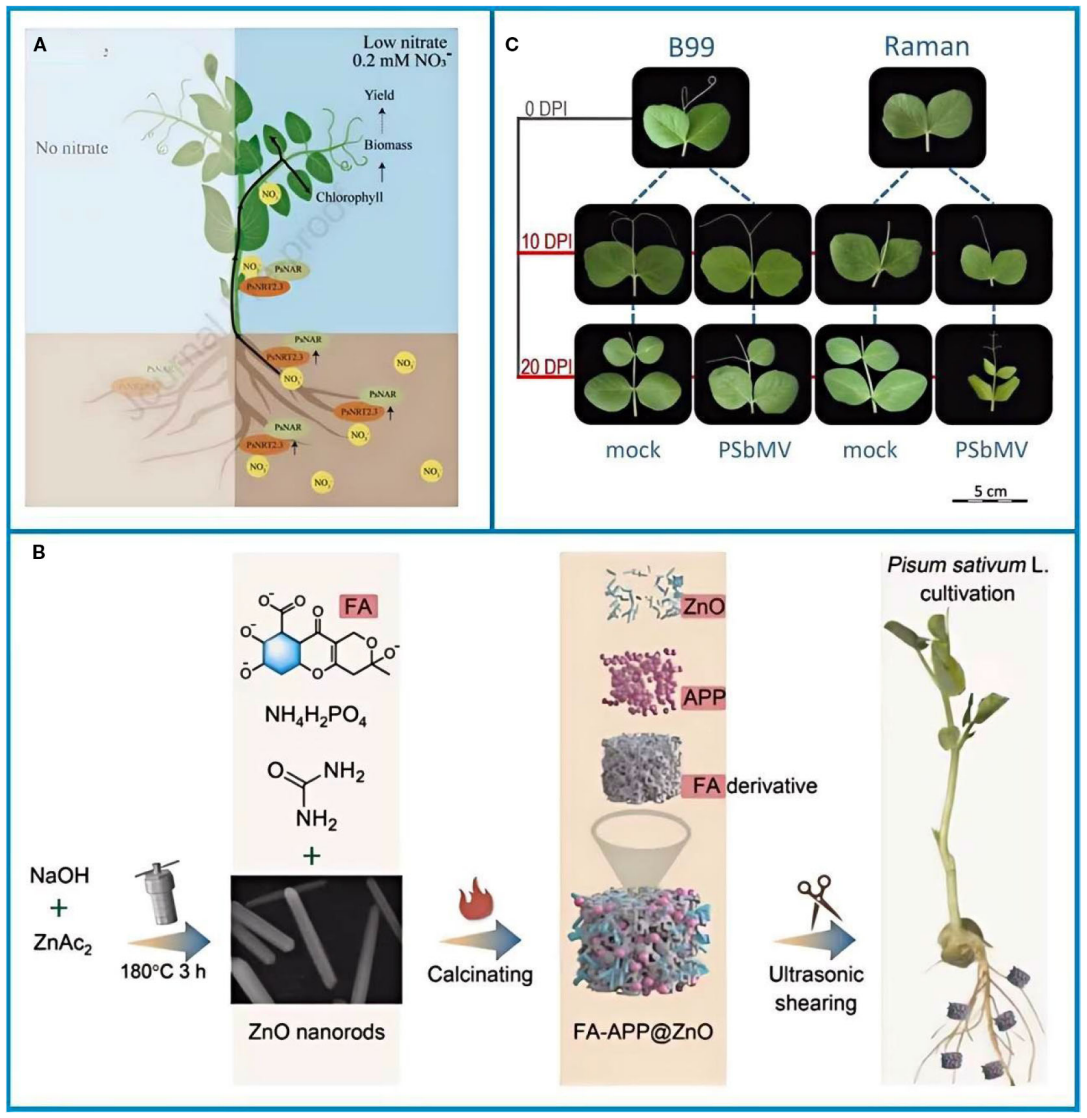
**(A)** A proposed model for the regulation of nitrate uptake in peas by PsNRT2.3 and PsNAR (Chen et al., 2024). **(B)** Schematic of the synthesis of FA–APP@ZnO and its application in pea cultivation (Han et al., 2022). **(C)** PSbMV-sensitive and resistant pea cultivars Raman and B99 were inoculated and harvested 10 and 20 days post inoculation (Cerna et al., 2017). The figure was reproduced from Ref (Cerna et al., 2017; Han et al., 2022; Chen et al., 2024). with permision from the rightsholderA.

Pea nutrient requirements change with each growth stage (**Figure 5A**) (Chen et al., 2024). In early growth, nitrogen (N) is crucial for developing strong roots and shoots. Although peas can fix their own nitrogen, a small application of starter N (5–8 kg/ha of urea) at the seedling stage helps the crop establish in low-fertility soils. Recent genetic research shows that the PsNRT2.3 gene helps regulate nitrate transport; cultivars with high expression of this gene can increase N uptake by 20–30% in nitrate-limited soils (Chen et al., 2024). The demand for phosphorus (P) is highest during flowering. At this stage, applying zinc (Zn) alongside phosphorus is critical to prevent P-Zn antagonism, a condition where high levels of one nutrient inhibit the uptake of the other (Ejaz et al., 2020; Han et al., 2022). Nano-fertilizers such as FA–APP@ZnO (**Figure 5B**), which co-deliver P and Zn via a fulvic acid–ammonium polyphosphate matrix, increase P and Zn uptake by 54% and 400%, respectively, compared to conventional fertilizers (Han et al., 2022). Potassium (K) is vital post-flowering; foliar spraying of 0.3% KH_2_PO_3_ at 30 days after flowering enhances pod filling and stem strength, reducing lodging by 15–20%.

#### 4.2.3 Water management

Water management for peas requires a careful balance, as the crop is sensitive to both drought and waterlogging. During critical growth stages, from budding to pod formation, irrigation should keep soil moisture at 70–75% of field capacity. If moisture in the top 20 cm of soil drops below 60%, drip irrigation at a rate of 30–40 m³/ha is recommended. To prevent root rot, it is essential that fields are drained within two hours after heavy rainfall. In arid regions, regulated deficit irrigation (RDI) during vegetative stages can improve water-use efficiency by 12–18% without yield loss.

#### 4.2.4 Integrated pest and disease control

Integrated strategies are needed to manage key pests and diseases. Pea seed-borne mosaic virus (PSbMV), for example, can cause yield losses of up to 40%. To reduce transmission risk, farmers can combine resistant cultivars (like B99) with a seed treatment of 10% trisodium phosphate for 20 minutes (**Figure 5C**) (Cerna et al., 2017). Aphids, which can spread the virus, can be managed with silver-reflective mulches and botanical insecticides, such as a 5% eucalyptus oil emulsion applied when aphid counts exceed five per plant. Pea weevils (*Bruchus pisorum*) are controlled via 40-mesh insect nets during podding and parasitoid wasps (*Anisopteromalus calandrae*), achieving 60–75% larval parasitism. Regular field monitoring and removal of infected plants minimize pathogen reservoirs.

## 5 Development of mechanized pea harvesting technology

With the development of modern agricultural technology, the mechanised harvesting technology of agricultural products is gaining importance internationally (Liang et al., 2016; Xu et al., 2016; Chen et al., 2017; Ji et al., 2017; Li et al., 2017; Zhao et al., 2018). In recent years, the pea industry has been developing rapidly, and the research and development of supporting harvesting machinery has become the most important link in the process of promoting the modernisation of the pea industry (Wei et al., 2018; Liang et al., 2019; Pang et al., 2019; Xu et al., 2019b; Xu et al., 2019a; Yang et al., 2019; Zhang et al., 2019). On the basis of the previous chapters, this chapter takes the actual situation of pea production and planting as the starting point, to clarify the characteristics of pea harvesting machinery to complete the harvesting operation and the current development situation.

### 5.1 Evolution of harvesting technologies

The mechanization of pea harvesting originated in the late 19th century with manual pod threshers, exemplified by Madame Faure’s pioneering device demonstrated at the 1885 Paris Exhibition. These early prototypes established the fundamental principle of mechanical shelling through rotational impacts. A significant leap occurred in the 1950s with mobile threshers featuring auto-leveling drums that maintained operational stability on uneven terrain.

The 1970s marked a technological watershed through the introduction of multi-beater systems. By replacing single-impact drums with five sequentially arranged beaters, this innovation reduced pea damage by 40% through gradual pod opening compared to conventional high-impact methods. Contemporary advancements focus on intelligent harvesting systems integrating automated adjustment (Chen et al., 2016; Lu et al., 2018; Umani et al., 2020; Hu et al., 2022), real-time loss monitoring (Ouyang et al., 2016; Hu et al., 2017; Zhang et al., 2017; Lian et al., 2021; Liang, 2021; Li et al., 2022; Wu et al., 2023; Guo et al., 2025), and multi-crop compatibility (Jiang et al., 2017; Zhai et al., 2017; Wang et al., 2019; Zhang et al., 2019; Wang et al., 2020; Sharma et al., 2021; Ji et al., 2022). For instance, modern harvesters (Benin, 2015) like the Dutch EPD540 series (Kumar et al., 2019) achieve complete pod separation and straw crushing through optimized drum kinematics and sensor-based speed modulation, demonstrating 50-fold efficiency gains over manual harvesting (Salawu et al., 2001; Beres and Husti, 2010; Karagic et al., 2010).

Similarly, research on pea harvesting machinery began in the United States in the 1970s and 1980s. The 2430 multifunctional harvester produced by the United States Ten International Company can be used for harvesting different crops such as peas and leafy vegetables. This high-efficiency, intelligent harvesting machinery greatly reduces the burden of labour and improves the quality of harvesting when harvesting peas, and plays an important role in promoting agricultural development and increasing farmers’ incomes.

### 5.2 Characteristics of modern pea harvesting machinery

Peas are well known internationally as an important food legume (Sarkar et al., 2020) and animal protein feed. As the development of the times, the labour force is decreasing and the use of machines instead of manual labour is gradually developing as a trend in order to ensure the efficiency of crop production (Chai et al., 2020; Chen et al., 2020; Liang et al., 2020; Xu et al., 2020; Ding et al., 2022; Liang et al., 2022; Li et al., 2022; Luo et al., 2022; Cong et al., 2023; Huang et al., 2023; Liang and Wada, 2023). Such trends have also accelerated the shift towards mechanisation and automation in international agriculture. In addition, the expansion of the scale of pea cultivation has led scholars to design a special shelling mechanism for the characteristics of pea pods and pea seedlings (Pérez-Petitón et al., 2018; Chai et al., 2020; Selvan and Mani, 2020). However, the degree of mechanisation is extremely low, primarily due to a combination of high economic costs, technical bottlenecks related to crop lodging, diverse topographies, and the prevalence of smallholder farming systems. These factors represent the main reasons restricting the further development of the pea planting industry.

Environmental adaptability constitutes another critical consideration (Rubiales et al., 2019). Pea cultivation spans diverse topographies from flat plains to mountainous regions, demanding harvesters that reconcile operational efficiency with terrain flexibility (Hassan et al., 2021; Pan et al., 2022; Duan et al., 2023; Zhang et al., 2023; Lakhiar et al., 2024; Xu et al., 2024). While large combine harvesters achieve high throughput in plains, their bulkiness renders them unsuitable for sloped fields. Conversely, compact machinery designed for mountainous areas often compromises harvesting capacity when deployed in expansive flat fields. This paradox underscores the need for modular designs accommodating adjustable working widths and terrain compensation systems (Selvan and Mani, 2020).

Economic viability further shapes harvesting technology development. The capital intensity of specialized equipment must be balanced against labor cost savings, particularly for smallholder farmers. Strategic use of universal components and localized manufacturing has emerged as essential for maintaining affordability while ensuring technical performance (Boateng and Yang, 2021a; Boateng and Yang, 2021b; Gao et al., 2021; Hou et al., 2023; Shi et al., 2023; Shah et al., 2024; Wu et al., 2024; Yang et al., 2024; Zhao et al., 2024; Liu et al., 2025).

### 5.3 Terrain-specific harvesting solutions

**Figure 6:**
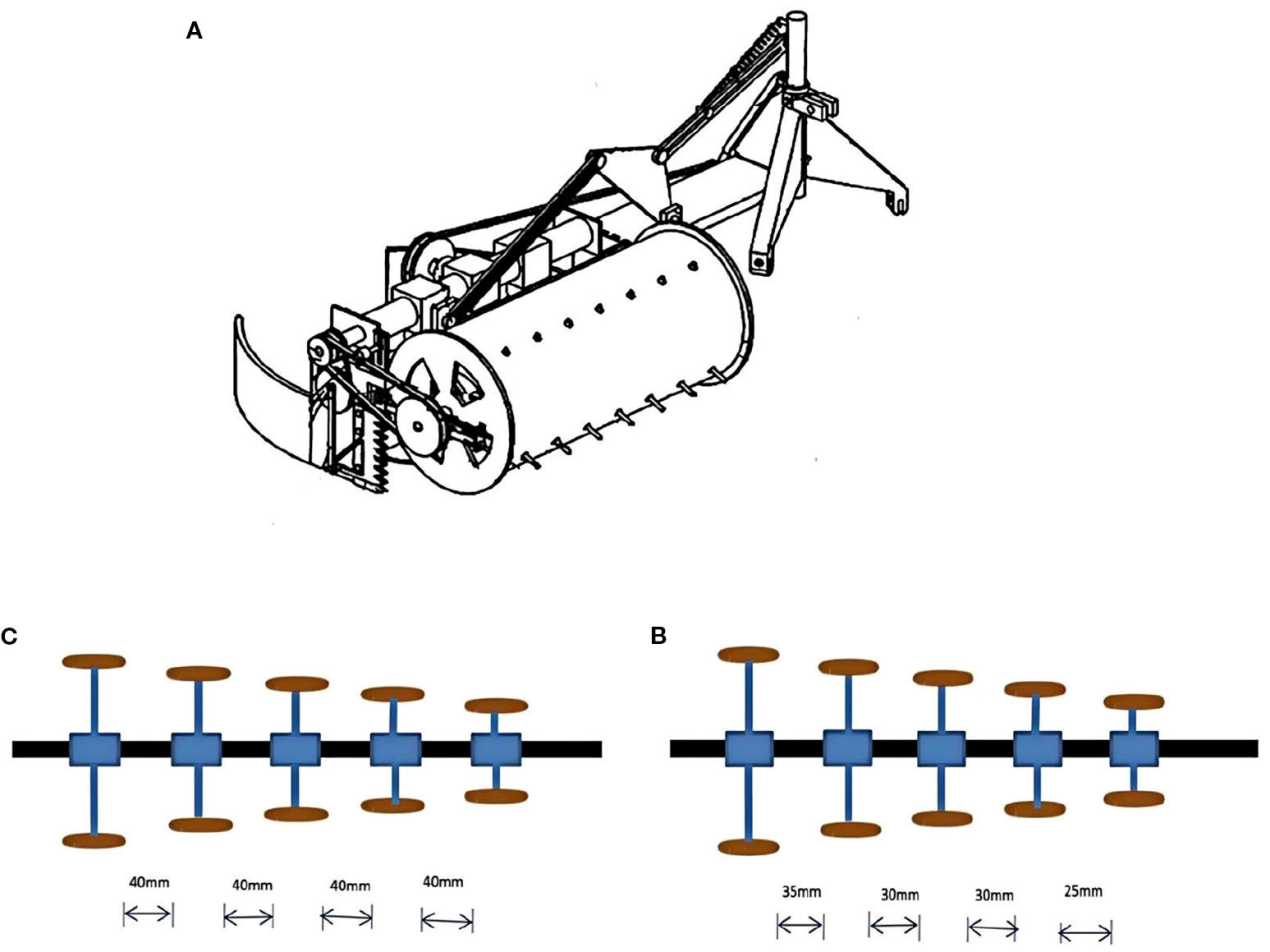
**(A)** The disc type pea cutting dryer (Jiyun et al., 2020). The gap between conical sieve and hammer pad: **(B)** the first setup, **(C)** the second setup (Kumar et al., 2021). The figure was reproduced from Ref (Jiyun et al., 2020; Kumar et al., 2021). with permission from the rightsholder.

However, large-scale harvesting machinery such as the EPD540 harvester is only suitable for harvesting in farm environments, and is extremely unsuitable for cultivation environments such as mountainous and hilly areas, and the high cost of such large-scale harvesting machinery manufacturing and service costs are difficult for individual farmers to afford. Mountainous and hilly cultivation areas demand specialized engineering solutions. A key trend in the literature is this technological bifurcation. Primary technical barriers include frequent machine clogging from entangled lodged plants and premature wear from ground debris (Coradi et al., 2022). The side-mounted disc harvester (**Figure 6A**), developed primarily in European contexts, addresses these challenges with features like pivoting drum units and anti-winding blades, reducing losses from 35% to 12% in sloped fields (Jiyun et al., 2020). In contrast, while effective, the capital cost and scale of such machinery present adoption barriers for smallholder farmers, who dominate pea production in the highland regions of Asia and parts of Africa.

Consequently, a consistent theme in recent research is the development of lightweight, modular harvesters. These machines (<800 kg), featuring high-strength steel frames and hydraulic self-leveling systems, are designed for flexibility on slopes up to 25°. However, a notable contradiction remains: while these lightweight solutions improve accessibility, their harvesting efficiency and durability often do not match their heavier counterparts, highlighting an unresolved trade-off between adaptability and performance. Future innovation must therefore focus on bridging this gap with cost-effective, robust designs tailored to smallholder economic realities.

Structural innovations further enhance terrain adaptability. Lightweight harvesters (<800 kg) with high-strength steel frames and hydraulic self-leveling systems maintain stable operation on slopes up to 25°. Debris resistance is improved through tungsten-carbide cutting edges (HRC 55-60) that withstand abrasive soil conditions, extending maintenance intervals to 200 operational hours. Despite these advances, current prototypes still struggle with extreme slopes (>30°) prevalent in Asian highland farming systems, highlighting the need for continued innovation.

Beyond pure technical performance, significant socio-economic barriers hinder the adoption of these advanced harvesters. The primary obstacle, particularly for smallholders who constitute a large portion of global pea producers, remains the high capital investment and overall affordability. Moreover, ongoing maintenance costs, the availability of spare parts, and the need for skilled operator training pose additional, often overlooked, challenges in rural contexts. While lighter machines improve terrain adaptability, a persistent trade-off often exists between agility and harvesting efficiency or durability. Therefore, future development must not only focus on technological innovation but also on creating economically viable, user-friendly, and locally serviceable solutions to bridge the gap between technological potential and practical adoption.

In some poor areas, peas were still threshed manually. Manual removal of kernels from pea pods is a labour-intensive and time-consuming task, whereby one person can remove about 3-3.5 kg of kernels from pea pods per hour. A small manual green pea thresher was successfully produced (Kumar et al., 2021). The performance of the small manual green pea thresher was evaluated by varying the gap between the conical sieve and the hammer pad (**Figure 6B**). The first setup was shown in Figure The gap between conical sieve and hammer pad was maintained at 40 mm. The second setup (**Figure 6C**) the gap between the conical sieve and the hammer pad was 35 mm, 30 mm, 30 mm and 25 mm respectively. The test results suggested that the best results were obtained in the second setup compared to the first setup (96.75 per cent debris removal efficiency and 2.17 per cent damage rate).

With the advancement of technology, agricultural labour has become more and more expensive urgently requiring mechanised harvesting and hulling. Mbuvi et al (Mbuvi and Litchfield, 1994). evaluated the role of two pea hulling machines (Taylor rubber drum type and Sinclair-Scott rotary drum type) and a green pea combine (FMC combine) in hulling and harvesting of green soya beans. The Taylor sheller had a shelling efficiency of 95% and a seed damage rate of 3%. The hulling efficiency with the Sinclair-Scott huller was 77% and seed damage was 7%. Harvesting of harvested pods with the FMC combine resulted in 87% seed recovery and 10.8% seed damage. In addition, blanching of pods prior to shelling had a remarkable effect on shelling efficiency and seed damage.

With the overall modernisation of agricultural machinery increasing, the mechanised harvesting of peas is an inevitable trend of combining scientific and technological development with agricultural development, which has a great impact on the agricultural economy. This chapter summarises the current status of the development of pea harvesting machinery in the international arena, which is of great significance for researching the plant characteristics and harvesting conditions of peas, optimising the design of mechanical structures, improving the versatility of harvesting machinery, developing pea harvesting machinery in line with planting modes, and realising the mechanised harvesting of peas.

## 6 Storage requirements and preservation strategies for peas

While the growth stages of peas (e.g., germination, flowering, fruiting) influence their initial nutritional profile, postharvest storage conditions critically determine their final quality and commercial viability (Acquah et al., 2018). Key quality parameters—including vitamin C, sugars, chlorophyll, texture, and pest resistance—are highly sensitive to storage methods. This section systematically evaluates modern pea storage technologies, their comparative advantages, and practical limitations, with emphasis on preserving organoleptic and nutritional properties. The Comparative analysis of storage technologies was shown in **Table 2**.

**Table 2:** Comparative analysis of storage technologies.

Technology	Key mechanism	Advantages	Limitations	Typical loss reduction (vs. traditional)	Relative cost/Energy profile	Typical shelf-life
Low-Temperature Storage (0°C, 90-95% RH)	Reduces respiration rate and microbial growth	Maintains freshness, color, and texture Minimizes aldehyde accumulation (Ruan et al., 2024)	Energy-intensive (inferred) Requires humidity control (Pariasca et al., 2001)	~50-70% (fresh peas, quality loss)	Very High (continuous refrigeration)	3–5 weeks
Modified Atmosphere Packaging (MAP)	Alters atmospheric composition (low O_2_, high CO_2_) to slow metabolism	Reduces oxidation and weight loss Extends shelf life (Fraser et al., 2001)	Requires precise gas composition Limited scalability	~30-50% (fresh peas, quality loss)	Moderate (packaging material cost)	7–14 days
Hermetic PICS Bags	Creates a low-oxygen environment through insect/seed respiration	Eliminates insect pests without chemicals; Reduces weevil damage (Kar et al., 2016); Simple to use	Primarily for dry peas; Limited reusability; Higher initial cost than standard bags	>98% (dry peas, insect damage)	Low (Initial cost: ~$2-3/bag; no energy)	Months (for dry peas)
Microporous Polypropylene Packaging	Allows gas exchange to prevent anaerobic conditions while maintaining humidity	Maintains crispness and chlorophyll (Cantwell and Saltveit, 2013); Cost-effective; Reduces moisture loss	Less effective for long-term storage than MAP; Variable performance based on perforation count (Singh et al., 2005)	~20-40% (fresh peas, quality loss)	Very Low (simple perforated bags)	5–10 days
Freezing (-18°C)	Halts biological activity by turning water to ice	Excellent long-term nutrient retention (Bajcan et al., 2013); Preserves color and flavor well	Requires blanching pre-treatment; Potential texture loss upon thawing; High energy consumption	>90% (quality & nutrient loss over time)	High (energy for freezing & storage)	Up to 12 months

Several factors significantly impact the storage quality of peas, including the form in which they are stored. Research indicates that shelled peas generally store better than unshelled peas (Ram et al., 2021), possibly due to the physical weakening of peas that occurs post-shelling. Optimal storage conditions for peas involve maintaining a temperature of 0 °C and a relative humidity of 90-95%. Additionally, the transfer of assimilates between the pod wall and the seed during storage has a significant effect on pea quality. The concurrent occurrence of rapid mineral loss from the pod wall and mineral gain in the seed contribute to poorer storage outcomes. Furthermore, storing pods with seeds leads to faster respiration rates and a more rapid loss of glucose and sucrose compared to storing the hulls alone.

The taste and texture of peas at harvest are largely dependent on the maturity of the pods. Following harvest, quality can decline, with a loss of sweetness and crispness, accompanied by degreening and the development of a granular texture. These sensory attributes also impact consumer acceptance. Specifically, studies have shown that ascorbic acid content is positively correlated with sweetness and negatively with a “moldy” trait, whereas antioxidant capacity remains relatively stable during storage and is less correlated with sensory perception (Berger et al., 2007). These compounds also play a key role in preserving organoleptic attributes by protecting plant material from physiological deterioration.

Appropriate packaging plays a crucial role in maintaining pea quality during storage and transport. Research conducted by Elwan et al (Pariasca et al., 2001). found that MPPP12 polypropylene bags effectively maintained pea quality throughout storage and simulated shelf-life, exhibiting high scores for visual appearance, firmness, crispness and taste, as well as higher levels of chlorophyll, Vitamin C, and sugars. Similarly, Anurag et al (Anurag et al., 2016). demonstrated the effectiveness of microporous polypropylene bags with 12 micropore holes in maintaining quality during storage and retail.

Modified atmosphere packaging (MAP) has also been investigated for its effect on storage. A study focusing on shelled green peas demonstrated that the combination of MAP with 3–6 perforations (0.4mm diameter) under cold room conditions (4-10 °C, 90-94% RH) created a favorable in-package environment, reducing weight loss and color change compared to unsealed packaging (Anurag et al., 2016). This indicates that shelled green peas can be stored using MAP with a controlled temperature and humidity extending shelf life while maintaining quality and reducing cost.

**Figure 7:**
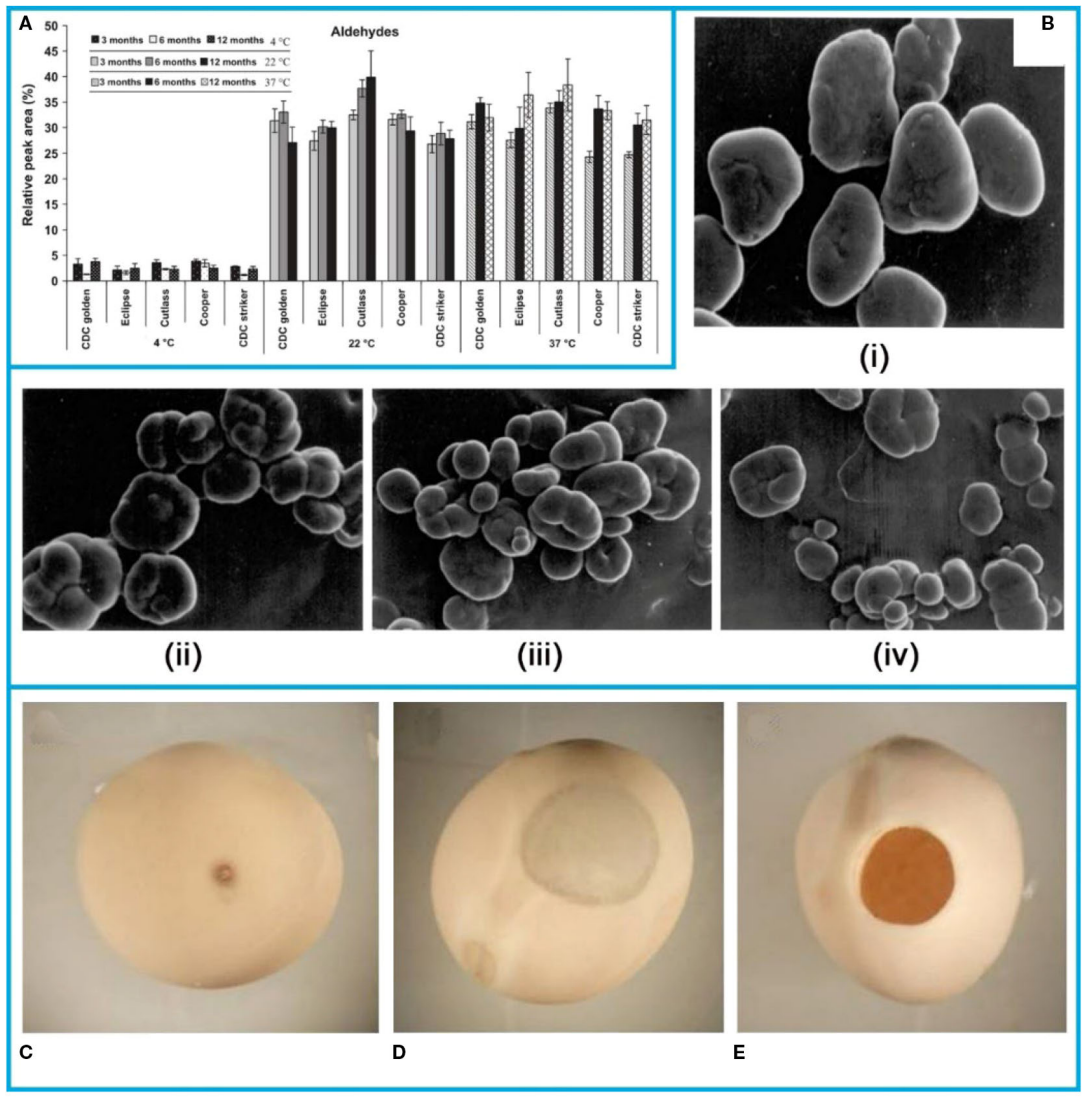
**(A)** Effect of storage time and temperature on the total aldehydes in pea cultivars (Azarnia et al., 2011). **(B)** Scanning electron micrographs (SEM) of starches separated from different pea cultivars (i) MA-6, (ii) VL-7, (iii) Arkel, (iv) NDVP-12 (Aggarwal et al., 2004). Damage symptom on pea grains caused by the pea weevil: **(C)** Sting, **(D)** window, **(E)** adult pea weevil exit hole (Mendesil et al., 2022). The figure was reproduced from Ref (Aggarwal et al., 2004; Azarnia et al., 2011; Mendesil et al., 2022). with permission from the rightsholder.

Several storage methods influence pea quality. Research has shown that temperature control during storage significantly impacts flavor. As shown in the **Figure 7A, a** study examining the volatile flavor profiles of pea seeds under different temperatures (4 °C, room temperature approximately 22 °C, and 37 °C) revealed that pea seeds stored at 4 °C had lower aldehyde content, which affect pea flavor, thus indicating that lower temperatures are favorable for storage (Azarnia et al., 2011).

Freezing is also an important technique for long term storage. While this method has minimal impact on nutrient content, it is important to be completed correctly. Studies have shown that storage condition influences phenolic compounds, that subsequently affects organoleptic properties (Ruan et al., 2024). During 10 months of storage at -18 °C, frozen peas exhibited smaller decreases in antioxidant activity but a larger decrease in total polyphenol content (Bajcan et al., 2013). In this respect, freezing is beneficial for long term storage of peas.

Furthermore, proper pretreatment with cryoprotectants like glycine betaine (GB) is important. While 5% GB did not provide sufficient protection, higher concentrations (10% GB) can maintain cell integrity and prevent damage caused by freezing (Kar et al., 2016).

The storage of crops in confined environments often leads to the unintended exposure of modified atmospheres (MA). Although not generally recommended, some studies show that peas can tolerate certain MAs. Testing showed that some MAs, such as 3% O_2_ + 6-7% CO_2_ and 10% O_2_ + 12% CO_2_ caused less damage on quality of the peas (Cantwell and Saltveit, 2013).

It was reported that the starch of legumes was sticky, which indicated that they were highly resistant to swelling and rupture, and therefore could be used as a raw material for a variety of industrial applications (Singh et al., 2005). The content of starch in peas determines the starting commercial value. The starch content of seeds of different pea varieties (MA-6, VL-7, Arkel and NDVP-12) was studied during storage (Aggarwal et al., 2004). It was observed that the larger the starch granules of seeds of different pea varieties during storage, the lower their starch content. The microscopic images of starch of different varieties of peas were shown in **Figure 7B**. The diameter of the granules (18.18-31.81 mm) of MA-6 pea starch was the largest as compared to other varieties.

Another major hazard affecting pea storage is storage pests, especially the pea weevil *Bruchus pisorum L.*, which causes significant losses. To address this, researchers have used Purdue Improved Crop Storage (PICS) bags. The main damage received by peas are spikes (**Figure 7F**), open windows (**Figure 7H**) and open holes (**Figure 7I**). To address the pest problem, three-layer sealed Purdue Improved Crop Storage (PICS) bags were used as a storage for peas and evaluated for storage potential.The results indicated that PICS bags can effectively maintained the post-harvest grain quality and reduced pea weevil damage by removing the need for insecticides on the stored peas (Mendesil et al., 2022).

Research has also evaluated the storage potential of other legumes in comparison to peas. For example, studies comparing peas with kidney beans (*Vicia faba*) showed that delaying the harvest of field legumes to 14 weeks reduced the storage potential of both but resulted in higher dry matter and crude protein yield, with field beans yielding higher amount than kidney beans. It was also noted that the storage potential of peas were significantly greater than that of kidney beans, when made into silage (Fraser et al., 2001).

For low-resource and smallholder settings, the choice between low-cost technologies like hermetic PICS bags and microporous polypropylene packaging is critical. Our synthesis indicates that these are not interchangeable. Hermetic PICS bags are highly scalable and effective for the long-term (months) storage of dry peas, offering superior protection against insect pests without chemicals, a key benefit for food security and market access. In contrast, microporous packaging is best suited for the short-term (days) storage and transport of fresh peas in regional supply chains, as it helps maintain crispness and color but offers limited protection against pests or long-term degradation. Therefore, the selection of the most appropriate scalable technology is highly context-dependent on the product form (dry vs. fresh) and the target storage duration.

The rapid growth of modern society, coupled with the demand from the processing and catering industries, has highlighted the need for effective and efficient methods for storing and transporting peas. The susceptibility of vegetables to quality deterioration post-harvest emphasizes the need for appropriate preservation measures in transport and storage, to minimize losses, and to prolong shelf life. To achieve these ends, it is imperative to understand the critical factors affecting pea storage. These include implementing appropriate pre-treatments, maintaining optimal temperature and humidity levels, using suitable packaging, and controlling atmospheric conditions, along with the effective management of pests. This chapter has analyzed these methods and characteristics, and thus provide useful insights into post-harvest technology of peas.

## 7 Conclusion

This review’s synthesis of pea agronomy reveals that the most critical barriers to sustainable production are systemic and interconnected, creating a “cascade effect” where inefficiencies in one stage compound problems in the next. To overcome these challenges, a paradigm shift from siloed optimization to a holistic, systems-based approach is essential. Future research should prioritize the following integrated directions:

1. Breeding for climate resilience and mechanization: Developing pea cultivars that not only exhibit tolerance to abiotic stresses like heat and drought but are also architecturally “machine-ready” (e.g., upright, shatter-resistant), directly linking genetic improvement to on-farm operational efficiency.
2. Farmer-centered, affordable mechanization: Designing and deploying scalable, low-cost harvesting and post-harvest technologies that are economically viable and serviceable in smallholder farming systems, moving beyond a one-size-fits-all model.
3. Digital agriculture integration: Leveraging sensor technology and data analytics to create feedback loops across the production chain—for instance, using real-time harvest data to optimize post-harvest storage conditions, thereby reducing waste and improving quality.
4. Risk assessment of emerging technologies: Conducting thorough, long-term assessments of the environmental (e.g., nanoparticle bioaccumulation) and socio-economic risks associated with new technologies to ensure their responsible and sustainable deployment.

Focusing on these integrated solutions is essential to bridge the gap between technological potential and on-farm reality, thereby solidifying the role of peas as a keystone crop for sustainable food systems.

Focusing on these interconnected research priorities is critical to unlocking the full potential of peas as a keystone crop for global food security.
